# Cardiovascular Disease Healthcare Utilization in Sub-Saharan Africa: A Scoping Review

**DOI:** 10.3390/ijerph16030419

**Published:** 2019-02-01

**Authors:** Herbert Chikafu, Moses J. Chimbari

**Affiliations:** School of Nursing and Public Health, College of Health Sciences, University of KwaZulu-Natal, Durban 4000, South Africa; chimbari@ukzn.ac.za

**Keywords:** burden of disease, cardiovascular disease, healthcare utilization, sub-Saharan Africa, utilization determinants

## Abstract

Sub-Saharan African (SSA) countries face a growing burden of cardiovascular disease (CVD), attributed to economic, nutritional, demographic, and epidemiological transitions. These factors increase the prevalence of CVD risk factors, and the CVD burden overlaps with a high prevalence of infectious diseases. This review aimed to understand CVD healthcare utilization determinants and levels in SSA. We conducted a systematic search of the literature on major databases for the period 2008–2018 using exhaustive combinations of CVD and utilization indicators as search terms. Eighteen studies from eight countries were included in this review. Most studies (88.8%) followed the quantitative methodology and largely focused on inpatient stroke care. Two-thirds of patients sought care within 24 h of suffering a stroke, and the length of stay (LOS) in hospital ranged between 6 and 81 days. Results showed a rising trend of CVD admissions within total hospital admissions. Coverage of physiotherapy services was limited and varied between countries. While few studies included rural populations, utilization was found to be negatively associated with rural residence and socioeconomic status. There is a need to extend healthcare provision in SSA to ensure access to the CVD continuum of care.

## 1. Introduction

Most low-to-middle-income countries (LMICs) are undergoing epidemiological transition. Improvements in healthcare provision and control of infectious diseases—notably HIV/AIDS in some countries in sub-Saharan Africa (SSA)—are increasing life expectancy and the ageing population [1]. This may increase the burden of non-communicable diseases (NCDs), as older people are more vulnerable [2]. Urbanization and lifestyle changes occurring in SSA are also predisposing young adults to NCDs, particularly cardiovascular diseases (CVDs) [3,4]. The rising prevalence of NCDs is overlapping with high levels of infectious diseases, including HIV/AIDS and tuberculosis, resulting in an unprecedented burden on the healthcare system.

Cardiovascular diseases pose major challenges to households and health systems. The World Health Organization (WHO) estimates that 40 million people die annually due to NCDs (about 70% of global deaths), with 75% of these deaths (28 million) occurring in LMICs [5]. Most NCD mortality (87%) is attributed to four major disease clusters: CVDs, cancer, respiratory diseases, and diabetes mellitus, with 45% (18 million deaths) of the NCD burden due to CVD. Furthermore, the burden of the leading CVDs and the prevalence of their risk factors is predicted to increase in LMICs [6]. Economic growth is also increasing the burden of CVD in SSA through the rising prevalence of risk factors. All other things being equal, income change often leads to lifestyle change, which promotes the consumption of unhealthy processed foods [7] and poses major health risks [8]. However, dietary change towards energy-dense food is also rising in rural and poor urban communities in SSA [8,9]. The income-related dietary changes in Africa have been found to be closely associated with major cardiovascular risk factors, hypertension, obesity, and diabetes [10,11,12]. While obesity exists in low-income communities [13], the nexus between income, obesity, diabetes, and hypertension is pronounced within and between African countries, with a higher prevalence of obesity and other CVD risk factors in higher-income communities [14,15]. CVD risk factors are almost enveloping all demographic groups in SSA, notably young adults [16] and school-going children [17]. The increase in risk factor prevalence across socioeconomic and demographic classes will increase the burden of CVD in SSA.

It is important to examine healthcare utilization for CVD in SSA in view of the rising prevalence. Health systems in most SSA countries are weak and centralized, with the majority of the population having limited access to healthcare services [18]. Healthcare services in Africa are also inequitable in favor of urban areas and households of high socio-economic status [19,20]. This, and other well-documented factors like distant health facilities, cultural barriers to healthcare access, the cost of care, and low health awareness [19], may affect health care-seeking practices for CVD. However, governments in SSA have committed to improving health outcomes through numerous programs, including the Sustainable Development Goals (SDGs), where the third goal (SDG 3) aims to ensure—among other things—universal health coverage (UHC) and a one-third reduction of premature mortality from NCDs through prevention and treatment by 2030 [21].

In this review, we aimed to understand health-seeking determinants and utilization levels for CVD in SSA. The historic association of NCDs with urbanization and affluence tends to concentrate studies on the NCDs in urban communities [13]. Consequently, information on CVD health services utilization in SSA is scant, despite the increasing burden of disease, particularly among poor and rural communities [3,22] where lifestyle change is increasing the exposure to CVD risk factors. Our study highlights the information gap on access to CVD healthcare services in SSA. The findings may instigate a review of CVD policy, financing, and service delivery in order to improve population health outcomes.

## 2. Materials and Methods 

### 2.1. Search Strategy and Selection Criteria

**Figure 1:**
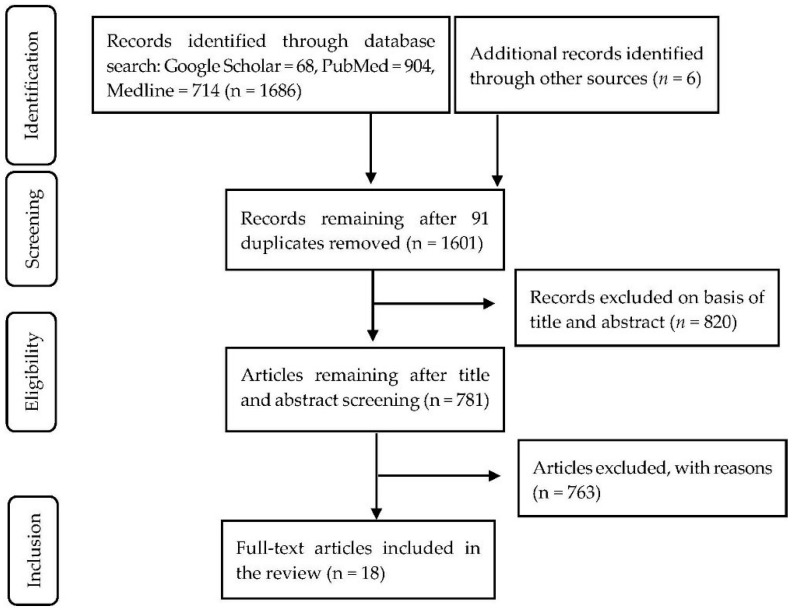
Study selection Preferred Reporting Items for Systematic Reviews and Meta-Analyses (PRISMA) flow diagram.2.2. Data Extraction and Synthesis.

We searched for literature on major databases (i.e., Medline, PubMed, and Google Scholar), limiting the search to articles published in English between September 2008 and September 2018 (See Supplementary File 1). The search used the AND and OR Boolean operators with exhaustive combinations of search terms for CVD such as cardiovascular, cerebrovascular, heart disease, vascular disease, and stroke; and terms for healthcare utilization indicators that included healthcare utilization, admission, inpatient care, outpatient care, hospitalization, and access to care. We also applied a country filter to limit our search to SSA. Following the removal of duplicate papers, articles not considering CVD health-seeking behavior and treatment were excluded based on title and abstract screening. Reference lists of selected papers were checked to identify studies for inclusion in the study. We followed the Preferred Reporting Items for Systematic Reviews and Meta-Analyses (PRISMA) guidelines (Figure 1).

### 2.2. Data Extraction and Synthesis

The following information was extracted from selected studies onto a template (Table 1): Publication details, country of study, objective(s) of the study, study design, sample size, and a summary of findings. For synthesis, extracted information was grouped into themes based on determinants, utilization categories, and levels. Utilization categories for the reported cardiovascular diseases specified the nature of health services received by patients that included inpatients are: Outpatient care, curative care, and restorative care. We also sought information on the extent of the unmet need for CVD, the quantum and distribution of care for respective cardiovascular conditions, and share of CVD within the overall hospital services rendered in a specific period.

## 3. Results

### 3.1. Overview of Selected Studies

We reviewed 18 studies from an initial collection of 1686 articles from Google Scholar, PubMed, Medline, and reference lists of searched articles (Figure 1). Reviewed studies examined CVD utilization in eight SSA countries: Nigeria (5), Ghana (3), Tanzania (2), South Africa (4), Ethiopia (1), Mozambique (1), Zimbabwe (1), and Rwanda (1). The majority (16) of studies followed quantitative methodology [23,24,25,26,27,28,30,31,33,34,35,36,37,38,39,40], while 2 studies employed qualitative [32] and mixed methods [29].

### 3.2. Healthcare Utilization

#### 3.2.1. Spectrum of CVD Care

Twelve reviewed studies examined stroke inpatient care [23,24,25,29,30,33,34,35,37,38,39,40], while the remainder reported on overall CVD [26,28,31,36], chronic care [32], and outpatient care [27] utilization. Besides stroke, acute coronary syndrome [26,31], cardiomyopathy [28], acute heart failure [26,28,31], peripheral heart disease [26], and arrhythmia [31] were also reported. Three studies assessed physiotherapy care as part of the integrated stroke continuum of care [24] and post-stroke inpatient and outpatient care [29].

#### 3.2.2. Utilization Indicators

CVD utilization was mainly reported as LOS in a health facility. Inpatient care for stroke ranged from mean LOS of 6 to 81 days. Five studies reported stroke inpatient care LOS of 6–17 days [23,24,25,33,39]. Only one study disaggregated LOS for ischemic stroke (median LOS = 38 days) and hemorrhagic stroke (median LOS = 81 days) [37]. Stroke inpatient care with physiotherapy services had higher LOS. A multi-country analysis including Rwanda, South Africa, and Tanzania reported a mean LOS range of 7.38 days (South Africa) to 12.6 (Tanzania) [29], while one study reported mean LOS of 16.2 days [34]. Six studies also reported CVD inpatient cases as a proportion of hospital admissions [24,26,27,29,30,32]. The share of CVD admissions increased considerably over two decades at facilities in Nigeria [28], Ethiopia [31], Ghana [39], and Tanzania [40].

Five studies examined the timeliness of accessing care among stroke patients [25,29,30,33,33]. At most, 60% of stroke patients presented at a health facility within 24 h time since stroke onset (TSO), while it took up to 14 days for other patients to receive care [25,30,33]. Assessment and referral for inpatient physiotherapy also varied considerably from 0.3 to 6.8 days TSO in South Africa and Rwanda, respectively [29]. However, referral rates were low in almost all studies that reported inpatient rehabilitation, except in South Africa, where there was almost universal referral [29].

Social profiles of study samples were mentioned in only 5 of the 18 reviewed studies [24,26,27,32,38]. Patients from rural areas [24,26,27,32] and from poor communities in both urban [38] and rural areas [24] accessed care for CVD. Socioeconomic status also determined access to care. In South Africa, stroke patients from a poor urban community received less or no physiotherapy services [38], while a poor rural community reported access barriers as strongly affecting their health seeking practices for chronic care [32].

## 4. Discussion 

We reviewed studies that assessed healthcare utilization for CVD in SSA. Overall, CVD utilization literature in SSA is sparse. The limited evidence and low CVD utilization levels are worrying in the backdrop of the rising prevalence of CVD risk factors, poor treatment and control of risk factors among the minority screened for risk factors, and the increasing burden of CVD in SSA [41,42]. The low utilization levels for CVD could be attributed to a number of factors, including poor knowledge and practices. Findings from a recent review study on CVD risk [43] and studies on CVD risk factors [44,45,46] showed poor awareness levels that likely cascade into the low CVD utilization reported in our study. Healthcare costs, which have been noted to hinder the utilization of chronic conditions, possibly contributed to the low utilization of CVD healthcare services reported in this review [32].

Health facility utilization indicators for CVD from reviewed studies suggest a notable level of efficiency in tertiary healthcare services and health systems in general. Mean LOS, a broad indicator of institutional efficiency [47], declined remarkably for stroke hospitalization [39], and compared favorably against other developing regions [26]. The decline in LOS could be attributed to improvements in hospital services, including the use of technology in the diagnosis and treatment [39] of stroke. On the other hand, an increase in the share of CVD admissions within total hospital admissions partially reflects improving access to healthcare services and health awareness in SSA.

However, the trend in LOS potentially reveals health policy shortcomings. The low LOS is possibly a result of cost containment measures, where health facilities limit hospital stay. Few studies reported physiotherapy as part of inpatient care for stroke patients, suggesting that physiotherapy is not generally provided as part of the inpatient continuum of care for stroke, and this reduces LOS. Most developing economies, including countries in SSA, implemented austerity measures in public expenditure as part of structural reforms in the 1990s, which reduced healthcare expenditure [48,49]. Notwithstanding improvements in healthcare provision and health awareness, the rising share of CVD admissions is associated with the increasing burden of CVD in the region. The ongoing epidemiological transition in SSA is increasing the prevalence of CVD risk factors and morbidity, hence the rising CVD admissions [50].

Reported utilization metrics provide limited evidence on health system performance with regards to CVD. None of the reviewed studies investigated whether healthcare utilization and institutional efficiency translated into health gains, which is the goal of health systems [51]. Furthermore, none of the reviewed studies differentiated LOS for stroke admissions by morbidity type, specifying whether it was an initial occurrence or a recurrence. Evidence shows varied recovery paths for stroke survivors being readmitted into hospital following an illness after discharge or suffering a recurrent stroke [52,53,54].

Findings show a worrisome trend in healthcare delivery in SSA. In spite of the increase in an aged population often vulnerable to chronic morbidity, studies suggest that there has not even been a proportionate increase in the supply of healthcare services, and more so for CVD [40]. This may explain the low utilization levels for CVD. As such, there is a need to expand and deepen the provision of healthcare services for CVD in order to address the unmet need, and as found in a study in South Africa [38], improve health outcomes and achieve health equity. Furthermore, universal coverage ought to be embedded in the provision of healthcare in view of the changing spatial and demographic prevalence of CVD and its risk factors.

Our study reports some limitations. We limited our review to studies published in English and possibly excluded a sizable number of studies in French and Portuguese, for instance, that met our inclusion criteria other than language.

## 5. Conclusions 

To the best of our knowledge, our study was the first to review CVD utilization in SSA. The literature on CVD utilization in SSA is limited, especially covering the quantification of socioeconomic and spatial differentials in utilization. Findings show the need for a more comprehensive investigation of access to CVD services from both demand and supply perspectives. Finally, rising CVD admission trends have implications for health policy in SSA. Primary health care provision needs to shift from a predominantly infectious disease approach to include NCDs. Importantly, the inclusion of preventive and rehabilitative care for CVD will strengthen the continuum of care, improve health outcomes, and help to reduce stroke recurrences and associated complications.

## Figures and Tables

**Table 1:** Summary of reviewed studies.

Reference	Country	Objective(s)	Study Focus	Study Design and Sample Size	Summary of Findings on Access to Care and Determinants
[23]	Nigeria	To understand length of stay (LOS) and determinants of stroke	Stroke inpatient care	Retrospective quantitative study (143 patients)	- 62% of patients were at least 40 years old, mean age was 61.5 years - Increasing stroke prevalence among young people - Most stroke burden came from ischemic stroke (53.8%) and hemispheric cerebrovascular disease (28%) - Mean LOS was 13.7 days with differences (not statistically significant) across stroke type and gender
[24]	Nigeria	Reviewing clinical patterns	Stroke inpatient care	Retrospective quantitative study (101 patients)	- Stroke had a 4.5% share of medical admission, 1.3% of hospital admission during the review period, and mean LOS was 12 days - Age of stroke patients ranged from 38 to 95 years (mean = 68 years), 49.5% of patients were at least 70 years old, and 47.5% of sample were male - 84.2% were from rural and low socioeconomic background - Most cases were ischemic (64.4) or intra-cerebral strokes (31.7%)
[25]	Ghana	To describe risk factors, clinical types, and stroke inpatient mortality	Stroke inpatient care	Observational, prospective quantitative study (265 patients)	- 56.6% of sample was female, mean age 64.6 years - 60% of stroke patients presented for care within a 24 h time since stroke onset (TSO), remainder presented between 2 and 14 days, most strokes were classified as ischemic (43%) and hemorrhagic (39%), mean LOS = 6 days
[26]	Tanzania	Estimating individual and household economic impact of cardiovascular disease	Cardiovascular disease (CVD) inpatient care	Retrospective, quantitative study (498 patients)	- Median LOS in Tanzania (5 days) was lowest in the study - Stroke accounted for 60.4% of CVD admissions in Tanzania, the remainder of admissions resulted from acute coronary syndrome (1.8%), acute heart failure (37.1%), and peripheral vascular disease (0.1%)
[27]	South Africa	Determining range and prevalence of reasons for attending outpatient care	All outpatient enquiries	Prospective cross-sectional survey, quantitative study (18,856 consultations)	- CVD was among conditions dominating ambulatory care consultations and increased with age - CVD consultations rose in ranking from fourteenth to third between 2001 and 2010
[28]	Ghana	Assessing CVD admissions and outcomes	Overall CVD inpatient care	Retrospective (11 years) quantitative (4226 patients)	- The share of CVD in total hospital admissions increased from 4.6% (2004) to 7.7% (2015) and heart failure admissions accounted for 88.3% of CVD admissions - Heart failure admissions came from hypertension (52.3%), cardiomyopathy (19.8%), and diabetes mellitus (10.3%)
[29]	South Africa, Rwanda and Tanzania	To assess post-stroke rehabilitation inpatient care services	Stroke inpatient care, rehabilitation	Retrospective, mixed methods surveys (South Africa = 168, Rwanda = 139, Tanzania = 145)	- Mean age ranged between 56.3 years (Rwanda) and 62 years (South Africa) - 98%, 67.5%, and 39.6% of patients received physiotherapy in South Africa, Tanzania, and Rwanda, respectively. LOS varied: South Africa—7.38 (+/−5.1), Rwanda—8.2 (+/−10.18), Tanzania—12.16 (+/−4.1). Admission TSO in days: South Africa (0.3), Rwanda (6.8), and Tanzania (1.2). Number of physiotherapy sessions was positively associated with LOS
[30]	Nigeria	To review patterns, types, and case fatality of stroke in young adults	Stroke inpatient care	Prospective quantitative study (71 patients)	- Mean sample age = 31.9 years (6+/−); 73% of sample was male; patient presentation TSO: within 6 h = 17%, 7–24 h = 32.4%, 25–48 h = 46.2%, >48 h = 4.2% - Classification: ischemic (59.2) and hemorrhagic (40.8%) stroke
[31]	Ethiopia	To describe trends of medical intensive care unit admission over 30 years in Ethiopia	CVD inpatient care	Retrospective quantitative study (500 patients)	- 57% of the sample was male, mean age = 40.2 years (+/−18), 42.6% of admissions resulted from CVD, CVD admissions were most prevalent in the 30–60-year age group - 35.7%, 29.1%, 17.4%, 6.1%, 4.7%, and 7% of CVD admissions were classified as acute coronary syndrome, heart failure, stroke, pulmonary thrombectomy, arrhythmia, and others - The share of CVD admissions increased from 21.7% to 58% over the study period, mostly from acute coronary syndrome, heart failure, and stroke, - Heart failure cases were high in the <30 year than in the >60 year age group
[32]	South Africa	Describing household experiences in accessing care for chronic illness in rural South Africa	Chronic care utilization	Household survey and qualitative longitudinal study(280 households)	- The study showed that access barriers—affordability, availability, and acceptability—influenced healthcare utilization for chronic care by households - While conclusions were not particular to specific chronic illness, there were self-reported and suspected cases of CVD
[33]	Mozambique	To assess incidence, characteristics, and short-term consequences of stroke hospitalization in Maputo	Stroke inpatient care	Prospective quantitative study (651 patients)	- 58.4%, 40.3%, and 1.3% of cases were classified as ischemic, hemorrhagic, and subarachnoid hemorrhage stroke, respectively - 60% of patients sought care within 24 h TSO, 21.5% after 1 day, 16.6% within 2–7 days, and almost 3% after 7 days - Mean LOS was 6 days
[34]	Nigeria	To investigate referral patterns and utilization of physiotherapy for stroke care at a hospital in Nigeria	Stroke inpatient physiotherapy	Four-year retrospective quantitative study (783 patients)	- Mean age of stroke survivors was 59.9 (+/−13) years, 42.2% of sample >=65 years, mean LOS was 16.2 (+/−12.3) days, 75.8% of stroke survivors were referred for physiotherapy with varying referral rates—71.4% (2010) and 81.1% (2012) - 35.3% of patients were assessed for physiotherapy within 24 h of admission, mean duration between admission and physiotherapy assessment was 3 days (+/−3.2), two-thirds of referred patients had in-patient physiotherapy - Utilization varied with age (elderly patients = 67.6%, below 46 years = 53.3) and gender (female = 67%, male = 59.8%), but not with type of stroke. - 63.4% and 25.2% of patients received inpatient and physiotherapy care respectively. Inpatient and outpatient physiotherapy sessions were equal (mean = 9), with shorter LOS among patients receiving physiotherapy - No significant association (*p* > 0.05) between LOS and age and gender, inpatient physiotherapy and age, gender and type of stroke
[35]	Zimbabwe	Description of clinical characteristics and outcomes of stroke patients in tertiary hospitals in Zimbabwe	Stroke inpatient care	Retrospective quantitative study (450 patients)	- 63% of stroke patients were female, 0.61% of 86,273 admissions were diagnosed with stroke, stroke admission rates ranged between 0.28% and 7.71%. - 54% and 46% of patients suffered hemorrhagic and ischemic stroke, respectively; CT scans were done on 39.4% of patients - LOS varied significantly across hospitals: 11.5 (+/−4) days, 7.8 (+/−6.3) days and 5.3 (+/−3) days. Overall mean LOS was 8.1 (5.7) days
[36]	Nieria	Determining differences in CVD mortality admissions between weekend and after-hours	CVD inpatient care	Three-year retrospective, quantitative study (339 patients)	- CVD admissions constituted 34.5% of medical admissions, 61% of patients were female, and the median age was 55 years - Stroke (55.2%) and congestive heart failure (42.5%) were the CVD conditions for admission, most (75.8%) admissions were during weekdays, most (54.4%) patients were admitted within 14 days
[37]	Rwanda	Determining the burden of stroke in Rwanda	Stroke inpatient care	Prospective observational quantitative study (96 patients)	- Mean age of patients was 59.7 years, age range: 19–91 years - The proportion of stroke in total admissions was 2.1%, comprising hemorrhagic (63.5%) and ischemic (36.5%) strokes - Median LOS was 38 days for ischemic stroke and 81 days for hemorrhagic stroke
[38]	South Africa	To determine survival, disability, and functional stroke outcomes following discharge from hospital	Stroke inpatient care	Retrospective observational quantitative study (196 patients)	- Median LOS was 8 days, 54.1% of strokes could not be classified due to lack of facilities, 37.2% and 8.7% were infarction and hemorrhage strokes respectively - 11 patients were discharged to inpatient rehabilitation, 45 patients with severe stroke were not considered for inpatient rehabilitation and were discharged home or to a care facility
[39]	Ghana	To assess stroke admission and mortality rates	Stroke inpatient care	Retrospective quantitative study (12 233 admissions)	- The share of stroke in total hospital admissions increased from 5.32 (1983) to 13.59/100,000 (2013), the mean age of patients increased from 58.9 (1983) to 62.3 (2013), mean LOS declined from 9 (1980s) to 6 days (2000s) - Unclassified stroke cases decreased from 66.9% (2008) to 54.4% (2013) due to improved imaging facilities
[40]	Tanzania	To assess stroke admissions to a tertiary referral hospital	Stroke inpatient care	Retrospective and quantitative (305 stroke admissions)	- Mean annual stroke admissions increased from 1.3 (1974) to 153 (2008) - Although regional population doubled during the study period, the number of hospital beds did not increase

## Supplementary Materials

The following are available online at http://www.mdpi.com/1660-4601/16/3/419/s1.
