# Supplementary material for: Cardiovascular Disease Healthcare Utilization in Sub-Saharan Africa: A Scoping Review
Source: Int J Environ Res Public Health. 2019 Feb 1;16(3):419. doi: 10.3390/ijerph16030419 (PMC6388380; doi:10.3390/ijerph16030419)
Supplement: Supplementary file 1 [file ijerph-16-00419-s001.pdf]

Supplementary File 1: Search Strategy.

Search Level	Search Terms
1	Cardiovascular OR Cerebrovascular OR Peripheral vascular events OR Vascular events OR Myocardial infarction OR Stroke OR CVA OR TIA OR Unstable angina OR Coronary heart disease OR Congestive heart failure OR Cerebrovascular disease OR Peripheral vascular disease OR Ventricular arrhythmia OR Arrhythmia OR Vascular interventions OR Thrombolysis
2	Healthcare utilization OR Utilization OR Health services OR Access to care OR Hospitalization OR Inpatient care OR Outpatient care OR Admission OR Length of stay OR LOS
3	Africa filter: Angola OR Benin OR Botswana OR 'Burkina Faso' OR Burundi OR Cameroon OR 'Cape Verde' OR 'Central African Republic' OR Chad OR Comoros OR Congo OR 'Democratic Republic of Congo' OR Djibouti OR 'Equatorial Guinea' OR Eritrea OR Ethiopia OR Gabon OR Gambia OR Ghana OR Guinea OR 'Guinea Bissau' OR 'Ivory Coast' OR 'Cote d'Ivoire' OR Kenya OR Lesotho OR Liberia OR Madagascar OR Malawi OR Mali OR Mauritania OR Mauritius OR Mozambique OR Namibia OR Niger OR Nigeria OR Principe OR Reunion OR Rwanda OR 'Sao Tome' OR Senegal OR Seychelles OR 'Sierra Leone' OR Somalia OR 'South Africa' OR Sudan OR Swaziland OR Tanzania OR Togo OR Uganda OR 'Western Sahara' OR Zambia OR Zimbabwe OR 'Central Africa' OR 'Central African' OR 'West Africa' OR 'West African' OR 'Western Africa' OR 'Western African' OR 'East Africa' OR 'East African' OR 'Eastern Africa' OR 'Eastern African' OR 'South African' OR 'Southern Africa' OR 'Southern African' OR 'sub Saharan Africa' OR 'sub Saharan African' OR 'sub Saharan Africa' OR 'sub Saharan African'
4	Limits: 30/09/2008 to 30/09/2018 in English and on humans
